# A case report of systemic lupus erythematosus with severe pulmonary hypertension presenting as large pericardial effusion with early signs of cardiac tamponade: a diagnostic and therapeutic challenge

**DOI:** 10.1093/ehjcr/ytae521

**Published:** 2024-09-20

**Authors:** Abdullah Ibrahim Alghamdi, Muhammad Azam Shah, Abdullah Mohammed Alkhodair

**Affiliations:** College of Medicine, King Saud University, King Khalid Road, Riyadh, Saudi Arabia; King Fahad Medical City, Dabab Street, Sulaimaniya, PO Box 221124, 11311 Riyadh, Saudi Arabia; King Fahad Medical City, Dabab Street, Sulaimaniya, PO Box 221124, 11311 Riyadh, Saudi Arabia

**Keywords:** Case report, Pericardial effusion, Cardiac tamponade, Systemic lupus erythematosus

## Abstract

**Background:**

Pulmonary hypertension is defined as resting arterial pressure >20 mmHg. Cardiac tamponade is a medical emergency where fluids accumulate in the pericardial sac compressing the heart pericardium leading to heart failure. Pericardiocentesis is challenging in patients with cardiac tamponade and severe pulmonary hypertension due to the risk of catastrophic haemodynamic collapse.

**Case Summary:**

An 18-year-old female who was recently diagnosed to have systemic lupus erythematosus (SLE) presented to the emergency department with shortness of breath, chest pain, fever, and fatigue. The physical examination revealed tachycardia, muffled heart sounds, and distended jugular venous pulse. Chest X-ray showed cardiomegaly, and transthoracic echocardiography showed a large circumferential pericardial effusion with signs of cardiac tamponade. There was severe pulmonary hypertension along with a dilated right ventricle with systolic dysfunction. The right ventricular systolic pressure was around 100 mmHg. The multidisciplinary team of cardiologists and pulmonologists decided to avoid pericardiocentesis due to the high risk of haemodynamic collapse. Aggressive medical therapy targeting pulmonary hypertension and SLE was started, which resulted in complete resolution of the pericardial effusion and normalization of pulmonary artery pressure.

**Discussion:**

A conservative approach can be an alternative strategy to manage patients with large pericardial effusion and impending pericardial tamponade in the presence of severe pulmonary arterial hypertension as pericardiocentesis carries a high risk of haemodynamic collapse.

**Learning points**

- Diagnosis of cardiac tamponade is challenging in the presence of significant pulmonary hypertension.
- Pericardiocentesis in patients with pulmonary hypertension can lead to significant haemodynamic collapse.
- Conservative therapy targeting pulmonary hypertension and aetiological causes can be an alternate management strategy in patients with large pericardial effusion and pulmonary hypertension.

## Introduction

Systemic lupus erythematosus (SLE) is a multisystemic autoimmune disease with diverse clinical presentations. The heart is one of the most affected organs in the SLE, and it can involve the pericardium, cardiac valves, myocardium, and coronaries, resulting in significant morbidity and mortality.^1^ Pericardial effusion and cardiac tamponade are rare initial presentations of SLE, and the standard treatment strategies include steroids and complete drainage for better cardiac outcomes.^2^ Systemic lupus erythematosus is commonly associated with pulmonary arterial hypertension (PAH), and its pathogenesis is multifactorial such as pulmonary arterial vasoconstriction, collagen deposition, and thrombosis of pulmonary circulation.^3^ The combination of large pericardial effusion and cardiac tamponade in the presence of severe PAH in patients with SLE poses a diagnostic and therapeutic challenge. The echocardiographic diagnosis of cardiac tamponade in the presence of significant pulmonary hypertension is difficult as such patients do not show classic signs of cardiac tamponade like right atrial or ventricular collapse due to high right-sided intracardiac pressures.^4^ Moreover, the drainage of pericardial effusion in patients with severe pulmonary hypertension is challenging due to concern of catastrophic haemodynamic collapse.^5^ In this case report, we present a young female who was recently diagnosed with SLE and she presented with large pericardial effusion and severe pulmonary hypertension.

## Summary figure

Transthoracic echocardiography (parasternal long-axis view) at presentation showing large pericardial effusion mainly around the inferolateral border of the left ventricle (white arrow) with diastolic collapse (yellow arrow) in (*A*) and presence of severe pulmonary hypertension as evident by continuous-wave Doppler (*C*). (*B*) and (*D*) show the resolution of pericardial effusion and normalization of right ventricular systolic pressure after treatment, respectively.

**Figure:**
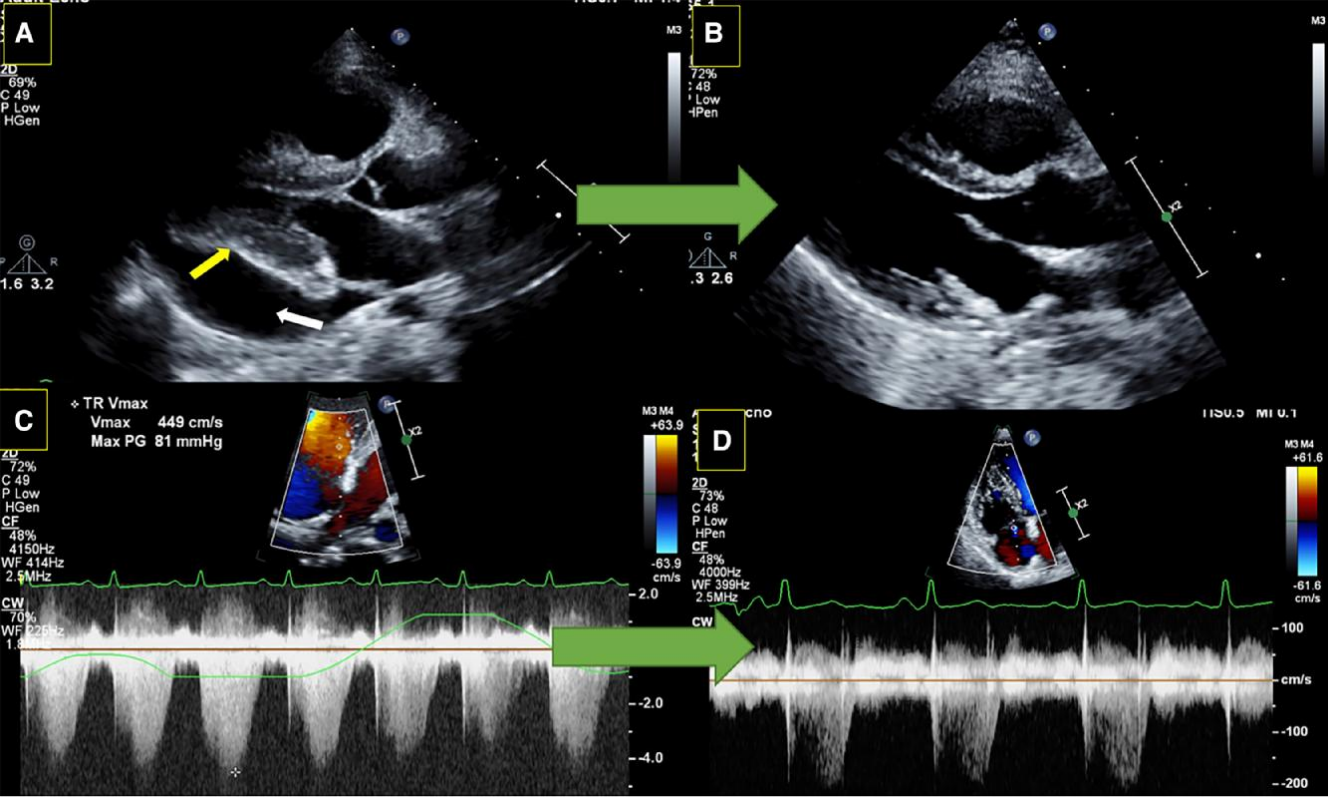


## Case presentation

An 18-year-old female who was recently diagnosed with SLE, presented to the emergency department with shortness of breath (New York Heart Association Class III) associated with palpitations and chest pain for 4 days. There was no history of any other medical or surgical illness. She also reported fever, fatigue, weight loss, joint pain, and a history of bluish discolouration of extremities with pain during exposure to cold (Raynaud phenomenon). She was diagnosed with large pericardial effusion and severe pulmonary hypertension in a secondary-level hospital and then referred to our centre (tertiary care) for further management. On admission, the patient was tachycardic with a heart rate of 142 beats per minute, a blood pressure (BP) of 106/68 mmHg, and a respiratory rate of 23 per minute. She was afebrile, and her oxygen saturation was 95% on room air. The cardiovascular examination revealed muffled heart sounds with a raised jugular venous pulse, and the respiratory examination was normal. She also had pedal oedema, and there were no clinical signs of peripheral hypoperfusion.

**Figure 1:**
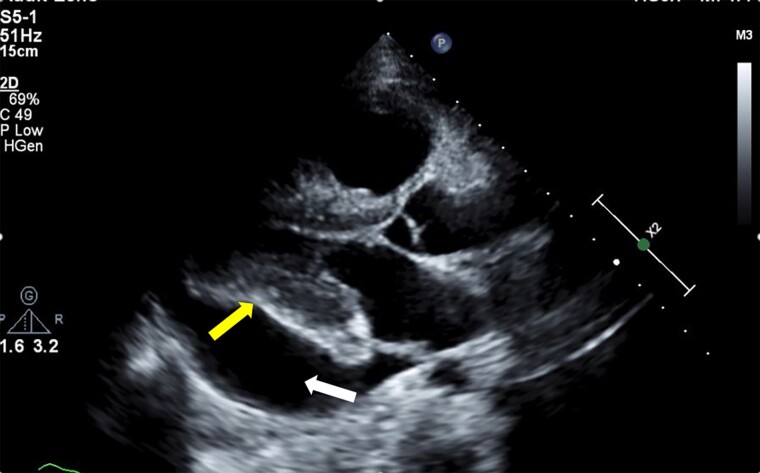
Transthoracic echocardiography (parasternal long-axis view) showing large pericardial effusion mainly around the inferolateral border of the left ventricle (white arrow) with diastolic collapse (yellow arrow).

**Figure 2:**
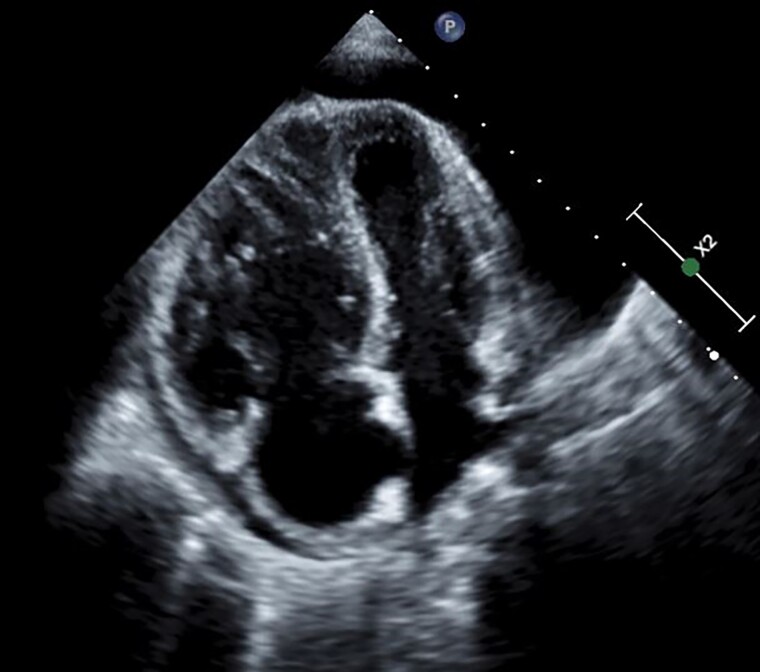
Transthoracic echocardiography (apical four-chamber view) showing large pericardial effusion and dilated hypertrophied right ventricle.

The electrocardiogram showed normal sinus tachycardia with non-specific ST-T changes in anterior chest leads. The chest X-ray showed cardiomegaly without signs of congestion. Transthoracic echocardiography showed a large circumferential pericardial effusion. The right ventricle and right atrium were dilated without collapse. However, the left ventricular diastolic collapse was noted along with significant respiratory variations across mitral and tricuspid inflow suggestive of cardiac tamponade. The right ventricular systolic pressure was around 90–95 mmHg (*Figures 1, 2,* and *3*, Supplementary material online, *Videos S1*, *S2,* and *S3*). The pulmonary trunk was also dilated. The right heart catheterization was performed and showed severe precapillary pulmonary hypertension with severely reduced cardiac output (*Table 1*). Her workup for SLE showed polyserositis as evidenced by raised inflammatory markers (erythrocyte sedimentation rate and C-reactive protein), and she also had positive dsDNA, antinuclear antibody, and low complement levels. The patient was admitted to the intensive care unit, and she was started on methylprednisolone sodium succinate 40 mg intravenously daily and hydroxychloroquine 200 mg daily orally for her SLE. Pulmonary hypertension management included riociguat 2.5 mg three times daily and macitentan 10 mg daily orally as intravenous therapies were not available. Pericardiocentesis was avoided, although the patient was showing echocardiographic signs of cardiac tamponade with borderline haemodynamic stability [tachycardia (>140 b.p.m.) and borderline blood pressure (systolic BP 100–110 mmHg)], due to a very high risk of haemodynamic compromise after pericardial fluid drainage in the presence of severe pulmonary hypertension.

**Figure 3:**
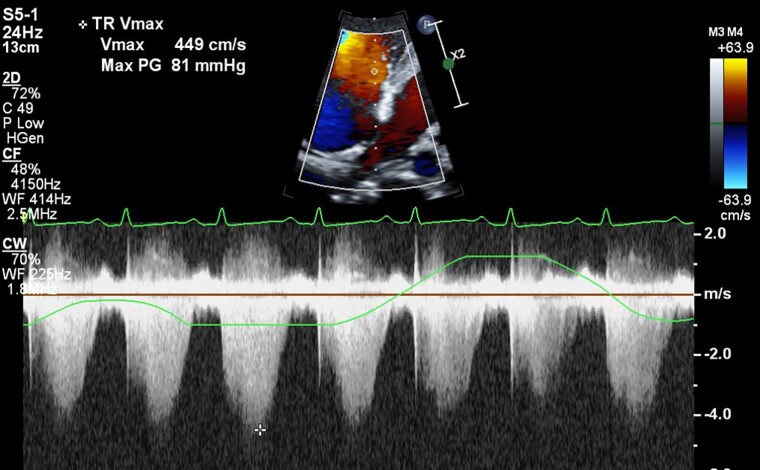
Transthoracic echocardiography showing continuous-wave Doppler across tricuspid valve showing a peak gradient of 81 mmHg.

**Table 1:** Right heart catheterization parameters at admission, 6 months, and 1 year

Parameters	At admission	After 3 months	After 6 months
Mean right atrial pressure (mmHg)	8	5	3
Right ventricle pressure (peak/diastolic/mean) (mmHg)	68\11\14	38\16\26	25/0/6
Pulmonary artery pressure (peak/diastolic/mean) (mmHg)	69/40/50	38/16/26	24/09/16
Pulmonary artery wedge pressure (mmHg)	8	8	7
Cardiac output (Fick method) (L/min)	1.94	3.3	4.4
Cardiac index (Fick method) (L/min/m^2^)	1.56	2.5	3.3
PVR (Woods unit)	23	5	2

**Figure 4:**
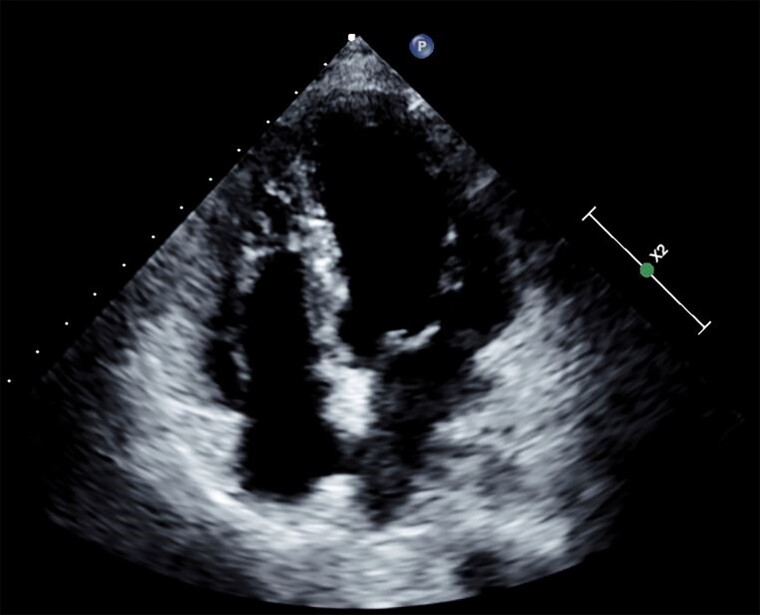
Transthoracic echocardiography (apical four-chamber view) showing complete resolution of pericardial effusion along with normalization of the right ventricular size.

After starting therapies for SLE and pulmonary hypertension, she showed a gradual improvement in haemodynamics and follow-up echocardiographies showed a significant reduction in pericardial effusion. She remained admitted for almost 1 month, and echocardiography at the time of discharge showed trivial pericardial effusion. After an initial stay in the intensive care unit, she was shifted to the cardiology ward and later discharged home. She was discharged on riociguat 2.5 mg three times daily, macitentan 10 mg daily orally for pulmonary hypertension, and hydroxychloroquine 200 mg daily orally, oral prednisolone for SLE. She was asymptomatic on follow-up in the outpatient department (at 1, 3, 6, and 12 months, respectively), and subsequent echocardiographies showed complete resolution of pericardial effusion along with normalization of the right ventricular size and function (*Figure 4*, Supplementary material online, *Videos S4* and *S5*). The repeated right heart catheterization studies conducted at 3 and 6 months showed a significant improvement in pulmonary artery pressures and cardiac output (*Table 1*).

## Discussion

Pericardial effusion can present as an incidental finding or as a manifestation of serious cardiac or systemic disease.^6^ Pericardial tamponade is a medical emergency that leads to low cardiac output and cardiogenic shock. Connective tissue diseases are one of the common causes of pericardial effusion. The presence of significant pericardial effusion along with PAH can be indicative of connective tissue diseases. Moreover, pericardial effusion and cardiac tamponade can be rare initial presentations of connective tissue disease like SLE.^1^ Pulmonary arterial hypertension elevates the right ventricular pressure, which will eventually lead to pericardial effusion by increasing pressure in the thebesian veins and coronary sinus.^4^ The management of cardiac tamponade in routine cases is percutaneous pericardial drainage, but this strategy cannot be applied in the presence of severe pulmonary hypertension and right ventricular dysfunction.^7,8^

Pericardial effusion in patients with PAH has been identified as an independent mortality risk factor. Management of such patients is not well established in the literature. Patients with pulmonary hypertension complicated by pericardial effusion currently carry a poor prognosis, and limited data exist to support management options in this complex clinical scenario.^9^ Recently, there has been a trend towards conservative management for patients with PAH complicated with pericardial effusion as pericardial drainage for these patients was associated with high mortality.^10^ The exact mechanism of poor outcomes is not well established. One possible mechanism of post-pericardiocentesis haemodynamic collapse is the rapid increase in preload to the diseased right ventricle which will fail to accommodate sudden high volume and pressure. Furthermore, the acutely distended right ventricle will cause compression to the left ventricle causing impaired diastolic filling of left ventricle further compromising cardiac output.

Case *et al.*^11^ reported that pericardiocentesis is safe and feasible in patients with severe pulmonary hypertension in a tertiary care set-up. The conservative management of small pericardial effusion in PAH is also reported, but the treatment of large pericardial effusion with signs of cardiac tamponade remains controversial.^12^ Our case represents the type of patient for whom there is no census on the management plan and delays in the decision can result in poor outcomes. Timely therapeutic interventions along with close monitoring are key to recovery. In our patient, conservative but aggressive management of SLE and pulmonary hypertension resulted in complete resolution of effusion and normalization of pulmonary artery pressures and right ventricular function.

In summary, a conservative approach can be an alternative strategy to manage patients with large pericardial effusion and impending pericardial tamponade in the presence of severe PAH in patients with SLE, as pericardiocentesis carries a high risk of haemodynamic collapse.

## Supplementary Material

ytae521_Supplementary_Data

## Data Availability

The data included in this article will be shared upon reasonable request to the corresponding author.

## Lead author biography

Dr Abdullah Ibrahim Alghamdi is a medical intern at King Khalid University Hospital. He graduated from the Riyadh College of Medicine in King Saud University with second-class honours with excellency. He is interested in internal medicine and interventional cardiology.

## Notes

**Consent:** The authors confirm that written consent for the submission and publication of this case report, including images and associated text, was obtained from the patient and family in line with COPE guidance.

**Funding:** None declared.
